# Tropism, pathology, and transmission of equine parvovirus-hepatitis

**DOI:** 10.1080/22221751.2020.1741326

**Published:** 2020-03-20

**Authors:** Joy Ellen Tomlinson, Mason Jager, Alyssa Struzyna, Melissa Laverack, Lisa Ann Fortier, Edward Dubovi, Lane D. Foil, Peter D. Burbelo, Thomas J. Divers, Gerlinde R. Van de Walle

**Affiliations:** a Baker Institute for Animal Health, Cornell University College of Veterinary Medicine, Ithaca, NY, USA; b Walnridge Equine Clinic, Cream Ridge, NJ, USA; c New York State Animal Health Diagnostic Center, Cornell University College of Veterinary Medicine, Ithaca, NY, USA; d Department of Clinical Sciences, Cornell University College of Veterinary Medicine, Ithaca, NY, USA; e Entomology Department, Louisiana State University, Baton Rouge, LA, USA; f Dental Clinical Research Core, National Institute of Dental and Craniofacial Research, National Institute of Health, Bethesda, MD, USA

**Keywords:** Theiler’s disease, serum hepatitis, horse fly, stem cells, vertical transmission

## Abstract

Equine parvovirus-hepatitis (EqPV-H) has recently been associated with cases of Theiler's disease, a form of fulminant hepatic necrosis in horses. To assess whether EqPV-H is the cause of Theiler's disease, we first demonstrated hepatotropism by PCR on tissues from acutely infected horses. We then experimentally inoculated horses with EqPV-H and 8 of 10 horses developed hepatitis. One horse showed clinical signs of liver failure. The onset of hepatitis was temporally associated with seroconversion and a decline in viremia. Liver histology and *in situ* hybridization showed lymphocytic infiltrates and necrotic EqPV-H-infected hepatocytes. We next investigated potential modes of transmission. Iatrogenic transmission via allogeneic stem cell therapy for orthopedic injuries was previously suggested in a case series of Theiler's disease, and was demonstrated here for the first time. Vertical transmission and mechanical vectoring by horse fly bites could not be demonstrated in this study, potentially due to limited sample size. We found EqPV-H shedding in oral and nasal secretions, and in feces. Importantly, we could demonstrate EqPV-H transmission via oral inoculation with viremic serum. Together, our findings provide additional information that EqPV-H is the likely cause of Theiler's disease and that transmission of EqPV-H occurs via both iatrogenic and natural routes.

## Introduction

Equine parvovirus-hepatitis (EqPV-H) was first described in 2018 in a case of Theiler’s disease, a form of acute hepatic necrosis [1]. In the United States, China, and Germany, EqPV-H has an estimated viremia prevalence of 2.9–17%, with 54% prevalence on farms where Theiler’s disease was recently documented [1–6]. EqPV-H infection is closely linked to both Theiler’s disease and subclinical hepatitis [2,7]. This is unusual among autonomous parvoviruses, because they typically require rapidly dividing cells to transcribe their viral genome [8]. In contrast, the liver is a terminally differentiated tissue with low basal levels of cellular division [9]. Therefore, it is essential to determine whether the tissue tropism of EqPV-H is compatible with its proposed pathogenicity.

Theiler’s disease, a.k.a. serum hepatitis or idiopathic acute hepatic necrosis, is commonly associated with administration of equine biologic products, but can also occur in horses that have not received such treatments [2,7,10]. With increasing evidence that EqPV-H is the causative agent of Theiler’s disease, it becomes of critical importance to understand how this virus is transmitted among horses. Iatrogenic transmission of EqPV-H has been clearly demonstrated via intravenous administration of equine origin blood products [1]. More recently, iatrogenic transmission via local administration of allogeneic bone-marrow-derived (BM) mesenchymal stromal cells (MSC) into tendon lesions has been suggested [7]. Still, iatrogenic transmission alone cannot account for the high prevalence of infection and the clinical cases of Theiler’s disease which occur without any preceding intended blood product inoculations [1,2,4,11].

The aim of this present study was, therefore, to evaluate tissue tropism and pathogenicity of EqPV-H in experimentally-inoculated horses, and to assess potential modes of transmission.

## Materials and methods

### Animals

Horses were 2–27 years old, 10 mares and 7 geldings, and 4 Warmbloods, 3 Thoroughbred crosses, 2 each of Thoroughbreds, Arabians, Morgans, Quarter Horses, and other breeds. Horses were confirmed (i) equine hepacivirus (EqHV, aka hepacivirus A, non-primate hepacivirus) serum quantitative PCR (qPCR) negative, (ii) EqPV-H serum qPCR negative, (iii) EqPV-H luciferase immunoprecipitation assay (LIPS) seronegative [1,12], and (iv) healthy by physical examination, complete blood count, and serum biochemistry including markers of liver disease and function before enrolment in the study. For assessment of vertical transmission, subjects were mares and foals on a Standardbred breeding farm (Farm E in a recent study [2]). All animal procedures were approved by the Cornell University Institutional Animal Care and Use Committee.

### Experimental procedures

#### Inoculation and monitoring of infection

Horses were inoculated with equine biologic products containing EqPV-H via multiple routes and with doses according to availability of source material and the method of preparation (see Supplemental Table 1 for details of procedures for each horse). All inocula were demonstrated infectious by successful infection of at least one recipient. Horses were monitored by weekly serum EqPV-H qPCR for at least 8 weeks. Horses that did not become viremic were challenge-inoculated by another route to confirm susceptibility. Once viremic, weekly serum biochemistry was performed past resolution of hepatitis (all liver biomarkers within reference range) or for a minimum of 14 weeks. Serology was performed before each inoculation, weekly from 2 to 10 weeks and then monthly. Transcutaneous liver biopsies were obtained from Horses A, E, and L during hepatitis as detected by serum biochemistry and from Horses H-J before inoculation, during hepatitis, and after recovery from hepatitis (Supplemental Table 1). Six horses (Horses A, H–J, L, M) had weekly nasal, oral, and faecal swabs collected to evaluate viral shedding. Six horses (Horses B-G) were euthanized and tissues collected from major organs using fresh gloves and sterile blades for each organ. Aliquots were snap frozen. Samples were stored at −80°C.

#### Assessment of vertical transmission

Serum was collected from 24 mares and foals at birth before the foal suckled. Serum was collected from the same foals in December of the birth year (7–10 months of age). Commercially available anti-*Rhodococcus equi* (*R. equi*) plasma was administered to all foals at birth and at 1 month of age. Aliquots of each lot of plasma used were also collected. Serum and *R. equi* plasma samples were tested by EqPV-H qPCR.

#### Allogeneic BM-MSC collection from donor horses and injection in recipient horses

Donor horses A and H were inoculated intravenously (IV) with 5.0 × 10^6^ genome equivalents (GE) EqPV-H in equine serum. MSC were collected when viremia was 2.7 × 10^6^ and 5.7 × 10^6^ GE/ml serum, respectively. Sternal bone marrow aspirates were collected into 10,000 international units (IU) of sodium heparin (Sagent Pharmaceuticals, India)/ml. BM-MSC were prepared by plastic adherence, as previously described [13]. Cells were cultured in media with 20% foetal bovine serum (MSC-FBS; Atlanta Biologicals Inc., Flowery Branch, GA, USA) or 20% autologous horse serum (MSC-AHS), which was EqPV-H qPCR positive. MSC were washed twice in phosphate buffered saline (PBS) and resuspended to 1 × 10^7^ cells per ml PBS or AHS. EqPV-H was quantified by qPCR. Horses L and M received MSC injections intra-articular (IA) in the medial femorotibial joint. Superficial digital flexor tendon (SDFT) lesions were induced in Horses C and K by collagenase gel injection, as previously described [14], two weeks before MSC were injected into the lesion under ultrasound guidance. Horse D developed an SDFT injury 9 weeks before MSC were injected into the lesion.

#### Horse fly trapping and feeding on horses

Donor horses E and I were inoculated IV with 5.0 × 10^6^ GE EqPV-H in equine serum. Flies were trapped using H-traps (Bite-Lite LLC, Bethel, CT, USA) baited with dry ice. Horse flies were subfamily Tabanidae, Genus *Hybomitra* (medium sized, eyes with 3 stripes), and Genus *Tabanus* (including “greenheads” with green eyes and a horizontal red stripe). To minimize horse pain during the experiment, Tabanus atratus, the very large black flies, were not used. Flies were temporarily housed in perforated plastic bags with moistened towels for humidity and sugar cubes for nutrition. Donors (Horses E and I) and recipients (Horses B, I and N) were sedated with xylazine (Bimeda-MTC Animal Health Inc, Cambridge ON Canada) during fly transmission attempts. Flies were captured in clear plastic containers and applied to 6 × 6-inch clipped areas on the backs of donor horses. Flies were carefully observed and interrupted mid-feed when the abdominal plates began to separate by sliding a laminated card between the horse and the containment cup. Feeding was confirmed by the presence of a blood spot on the horse after the fly was removed. Flies were transferred in the clear cup to the recipient within 15 min, placed on the recipient’s back and allowed to feed until repletion. Flies were frozen and stored at −80°C for EqPV-H qPCR.

#### Liver biopsy

Horses were sedated with xylazine IV. Ultrasound-guided transcutaneous liver biopsy was performed with a 14-gauge 11.4 cm Tru-Cut^TM^ biopsy needle (Medline Industries, Northfield IL, USA).

### Sample analysis

#### Quantitative polymerase chain reaction (qPCR)

Viral nucleic acids (NA) were extracted from serum, cell-free fluids, and swabs with the QIAmp Viral RNA Mini Kit (Qiagen, Germantown, MD, USA) and from tissues with the DNeasy Blood and Tissue Kit (Qiagen, Germantown, MD, USA), according to manufacturer’s instructions. Pre-enrolment serum samples were screened for EqHV by qPCR, as previously described [7]. Samples from the vertical transmission study, Horse A, and horse flies were analyzed at the Animal Health Diagnostic Center (AHDC), Cornell University, as previously described [7]. The remaining samples were processed with Bio-Rad iTaq^TM^ Universal SYBR® Green Supermix (Bio-Rad Laboratories, Inc., Hercules, CA, USA) and 2 µl of NA in a 20 µl reaction volume. Primers EqPV-H qVP1 F15/R15 (CACGGTCCCAGGACATTTAC/TCACAGATCGTCCCTACCAC) and EqB2M DNA qF/R (CAGCAGGCAAAGAAGAATCC/CTCTATCCCGTCACCACACC) were used at 0.3 μM. Serial dilution of plasmids containing EqPV-H VP1 and beta-2 microglobulin (*B2M*) amplicons were used as standards and had linear ranges of 10^1^–10^6^ GE/PCR reaction (corresponding to 2.14 × 10^3^–2.14 × 10^8^ GE/ml sample) for EqPV-H and 10^3^–10^6^ copies/PCR reaction for *B2M*. Tissue qPCR included at least 1 × 10^5^ *B2M* copies per reaction. Samples below the limit of quantitation (LOQ) were deemed positive if the Ct was more than 2 standard deviations below the no-template control and the standard deviation of the replicates was less than 1.0. EqPV-H quantity was normalized to cell count for tissues, volume for cell-free fluids, and per swab for swabs. Standard curves served as positive controls for qPCR, and no-template controls were included for each probe on each PCR plate.

#### Histopathology

Slide preparation and labelling was performed by the AHDC on a fee-for-service basis. Tissue sections were labelled with haematoxylin and eosin (HE), Masson’s trichrome, reticulin, and antibodies against the T cell marker CD3 (Leica, catalogue #PA0553, mouse monoclonal anti-human clone LN10), the macrophage marker Iba1 (Wako Pure Chemical Industries, catalogue #019-19741, rabbit polyclonal) and the B cell marker Pax5 (Leica, catalogue #PA0552, mouse monoclonal anti-human clone 1EW). All labels were applied to samples from Horses H-J. Samples from horses A-E, and L were labelled with HE and CD3 IHC only.

#### In situ hybridization (ISH)

RNAScope® 2.5 HD-Red ISH (Advanced Cell Diagnostics, Inc., Newark, CA, USA) was applied to formalin-fixed, paraffin-embedded (FFPE) liver biopsies. Twenty proprietary RNAScope® ZZ probes were designed to cover the 3001–4177 base pair region of the VP1 gene and a red chromogen was used. ISH was performed according to manufacturer’s instructions with a 30 min antigen retrieval step. Where indicated, samples were pre-treated with DNase-1 (Sigma-Aldrich, St. Louis, MO, USA) at 37°C for 30 min after the antigen retrieval step to detect viral RNA only. Antisense probe ISH (detecting sense DNA and mRNA) was performed on liver samples from Horses A-E and L. Samples from Horses H-J were also labelled with sense probes and with sense and antisense probes after DNase treatment.

#### Serum biochemistry and haematology

Serum biochemical analysis and complete blood counts were performed by the AHDC on a fee-for-service basis. Biochemical tests performed were: aspartate aminotransferase (AST), sorbitol dehydrogenase (SDH), glutamate dehydrogenase (GLDH), gamma glutamyltransferase (GGT), bile acids, total, direct, and indirect bilirubin, creatine kinase (CK), and triglycerides. Reference intervals were: AST, 222–489 U/L; SDH, 1–6 U/L; GLDH, 2–10 U/L; GGT, 8–33 U/L; Bile acids, 2–10 μmol/L; Total bilirubin, 0.5–2.1 mg/dL; Direct bilirubin, 0.1–0.3 mg/dL; Indirect bilirubin, 0.3–2.0 mg/dL; Creatine kinase, 171–567 U/L; and Triglycerides, 14–65 mg/dL.

### Serology

Anti-viral protein 1 (VP1) IgG antibodies were detected in serum by Luciferase Immunoprecipitation System (LIPS) assay, exactly as previously described [1].

### Statistical analysis

Friedman’s two-way analysis of variance was applied to determine if viral load was associated with tissue type in acutely infected horses. Two-tailed Fisher exact test was used to compare infection rates in foals that had received EqPV-H-contaminated plasma versus foals that did not. Statistics were performed in R Studio Version 1.0.136 (RStudio, Inc.). Significance was set at *p* ≤ .05.

## Results

### EqPV-H is hepatotropic

**Figure 1.**
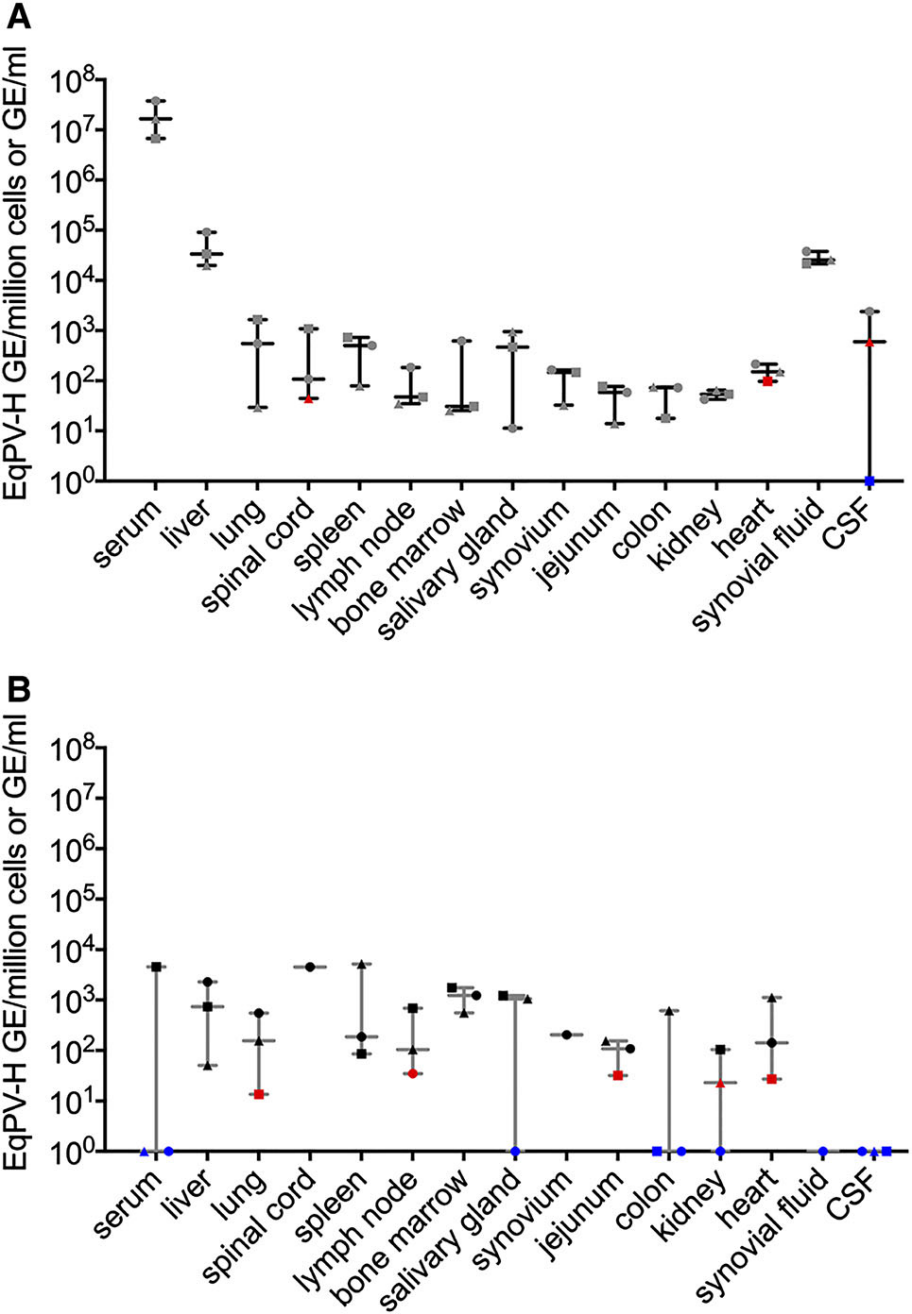
EqPV-H is hepatotropic and persists in tissues for months. (A) qPCR of tissues from 3 acutely infected horses at 5 weeks post inoculation and (B) three horses at least 15 weeks after infection. Viral load was significantly associated with tissue type in acutely infected horses (*p* = 0.028). Viral load was normalized to cell count by B2M qPCR for solid tissues or to volume for liquids. EqPV-H positive samples below the limit of quantitation are indicated in red, and samples below the limit of detection are indicated in blue.

To determine whether EqPV-H could be the causative agent of Theiler’s disease, the first step was to evaluate whether the virus is present in the liver. Horses B, C, and D were experimentally inoculated with EqPV-H and euthanized 5 weeks later, when viremia was high (>1 × 10^6^ GE/ml serum, Figure 1(A)). All three horses demonstrated highest viral load in the liver, although viral NA were also detected in other tested organs (Figure 1(A)). Viral load was significantly associated with tissue type (*p* = 0.028, Friedman’s two-way ANOVA).

Since other parvoviruses, such as human parvovirus B19, have viral NA persist in serum and tissues for months-to-years after infection [15–17], we assessed whether EqPV-H can establish persistence as well. Horses E, F, and G were euthanized at least 15 weeks after infection and showed persistence of viral NA in many tissues, similar to acutely infected horses (Figure 1(B)).

### EqPV-H experimentally-inoculated horses develop subclinical to clinical hepatitis

**Figure 2.**
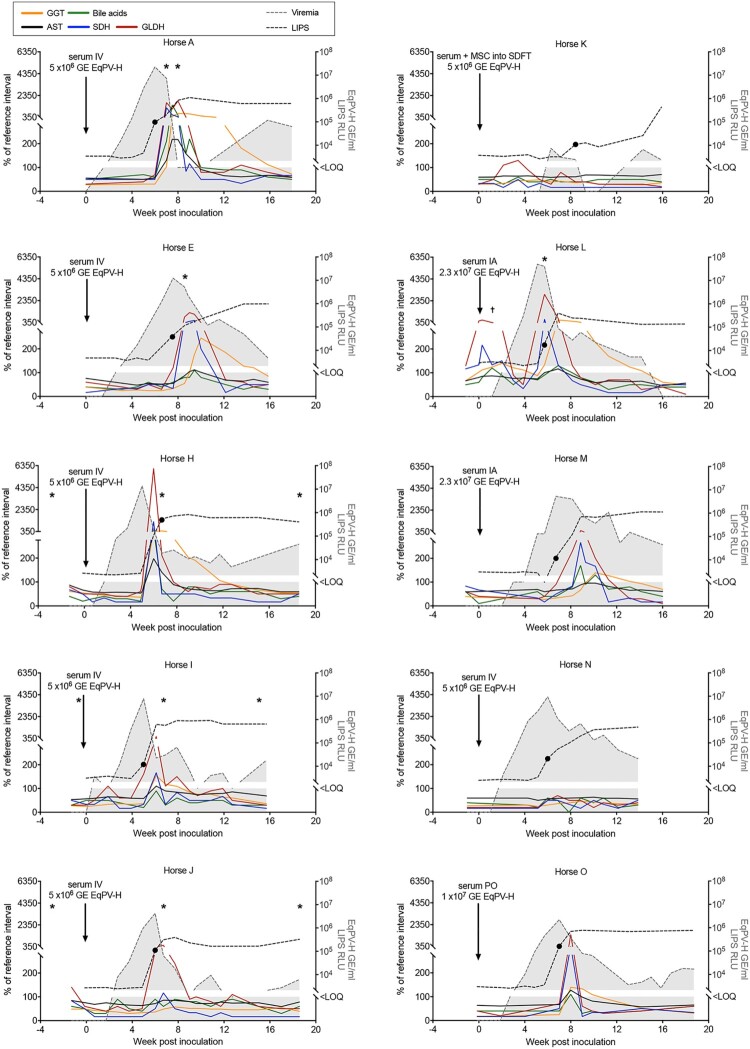
Serum biochemical, serologic, and virologic profiles of 10 horses experimentally infected with EqPV-H. Inoculation routes and doses varied and are indicated on each panel. *Times when liver biopsies were obtained. •First seropositive sample (positive cut-off 10^4^ RLU). ^t^Horse L was Equine hepacivirus (EqHV) negative when screened before inclusion in the study, however, she was naturally infected during the study and was found to be EqHV serum qPCR positive starting week −1 of IA EqPV-H^+^ serum inoculation. This was cleared by week 3, which was associated with a rise in liver enzymes, as is typical with EqHV infection. Liver markers were normalized to the maximum of the reference interval. Reference intervals: AST, 222–489 U/L; SDH, 1–6 U/L; GLDH, 2–10 U/L; GGT, 8–33 U/L; Bile acids, 2–10 μmol/L. GE, genome equivalents; EqPV-H, equine parvovirus-hepatitis; LOQ, limit of quantitation of PCR; RLU, relative light units for LIPS serology.

**Figure 3.**
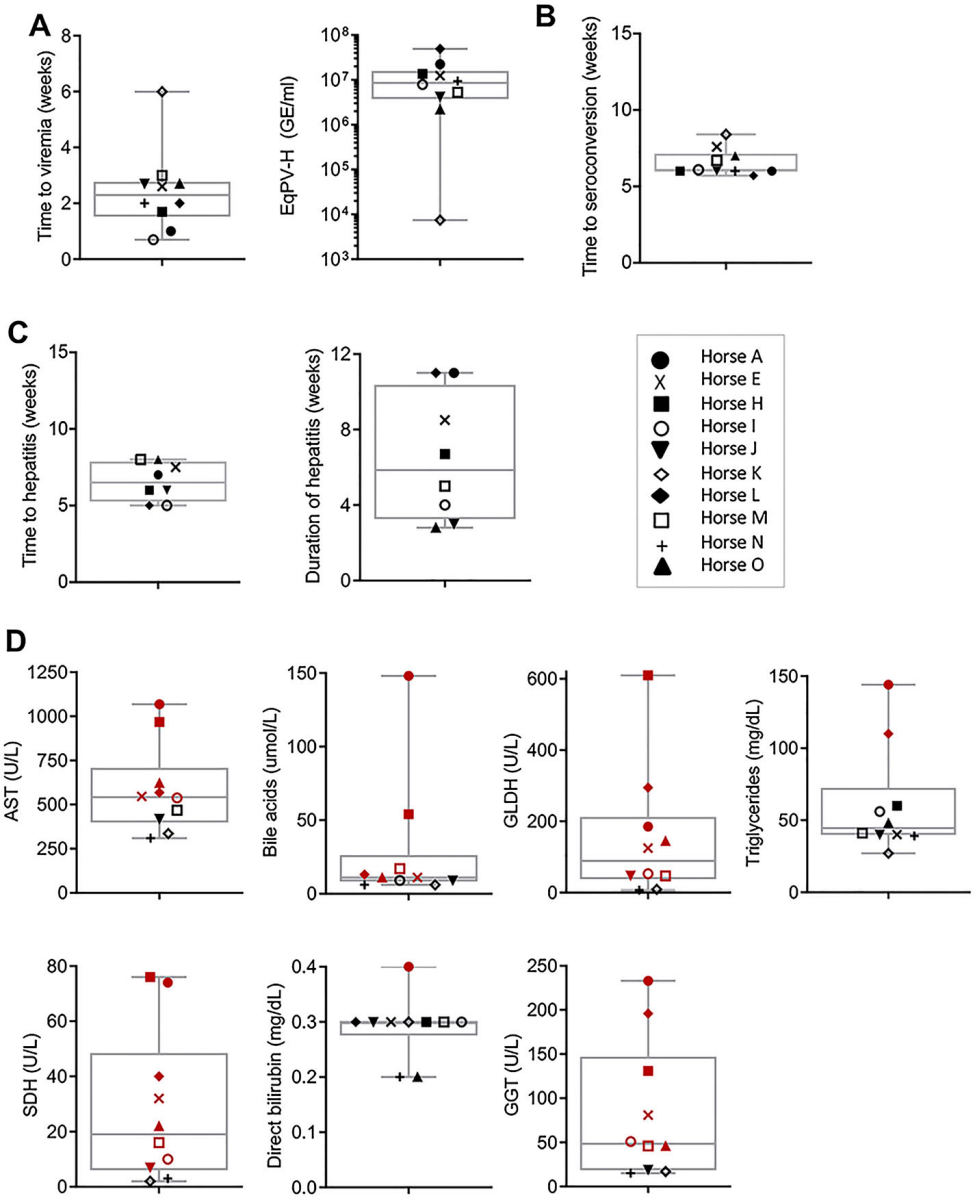
EqPV-H experimentally inoculated horses developed hepatitis at peak viremia. (A) Viral parameters (*n* = 10). (B) Seroconversion timing (*n* = 10). (C) Clinical parameters of horses that developed hepatitis (*n* = 8). Two horses did not develop hepatitis (Horses K and N) and one horse (Horse A) developed clinical signs of hepatitis including icterus, inappetence, and lethargy. (D) Peak serum biochemical markers (*n* = 10). Values above reference interval (RI) are indicated in red. RI: AST, 222–489 U/L; SDH, 1–6 U/L; GLDH, 2–10 U/L; GGT, 8–33 U/L; Bile acids, 2–10 μmol/L; Direct bilirubin, 0.1–0.3 mg/dL; Triglycerides, 14–65 mg/dL.

To further demonstrate the association between EqPV-H infection and hepatitis, serum biochemistry, serology, and qPCR were performed on 10 experimentally-inoculated horses (Figure 2). Viremia developed in a median 2.3 (range, 0.7–6.0) weeks after inoculation. Median EqPV-H at peak viremia was 8.6 × 10^6^ (range, 7.4 × 10^3^ – 5.0 × 10^7^) GE/ml serum (Figure 3(A)). Three of the 10 horses (Horses K-M) were followed to viral clearance, defined by 2 sequential negative serum qPCR, which occurred at 9, 16, and 45 weeks after inoculation, respectively. Interestingly, Horse K was again low positive on serum qPCR at 14 weeks after inoculation, despite having cleared by week 9, suggesting that viral DNA might have been persistent at levels around the limit of detection (Figure 2). Horses seroconverted in a median 6.0 (range, 5.7–8.4) weeks after inoculation (Figure 3(B)). Hepatitis, as determined by having at least 2 liver enzymes above reference interval, was detected in 8 of 10 horses, with a median time to hepatitis of 6.5 (range, 5–8) weeks and a median duration of 5.9 (range, 2.8–11) weeks (Figure 3(C)). Peak liver markers are shown in Figure 3(D). Onset of hepatitis occurred either the week of, or the week after, peak viremia and within one week of seroconversion in all 8 horses. Horse L showed increased liver enzymes 0–3 weeks after IA EqPV-H^+^ serum inoculation (Figure 2), which was associated with clearance of a naturally acquired hepacivirus infection (EqHV serum qPCR positive weeks −1–2). This horse had been screened EqHV negative both before the primary inoculation and 2 weeks before the IA serum inoculation. She was housed >75 yards from other horses with unknown viremia status. This EqHV-associated hepatitis resolved before Horse L developed a second episode of hepatitis co-incident with EqPV-H peak viremia and seroconversion. Horse A showed clinical signs of hepatitis, including icterus, mild lethargy and inappetence lasting for 6 days.

Collectively, these findings, particularly the close temporal association between peak viremia, seroconversion, and onset of hepatitis, indicate EqPV-H infection is associated with hepatitis.

### EqPV-H infects hepatocytes and results in hepatocellular necrosis and lymphocytic infiltrates

**Figure 4.**
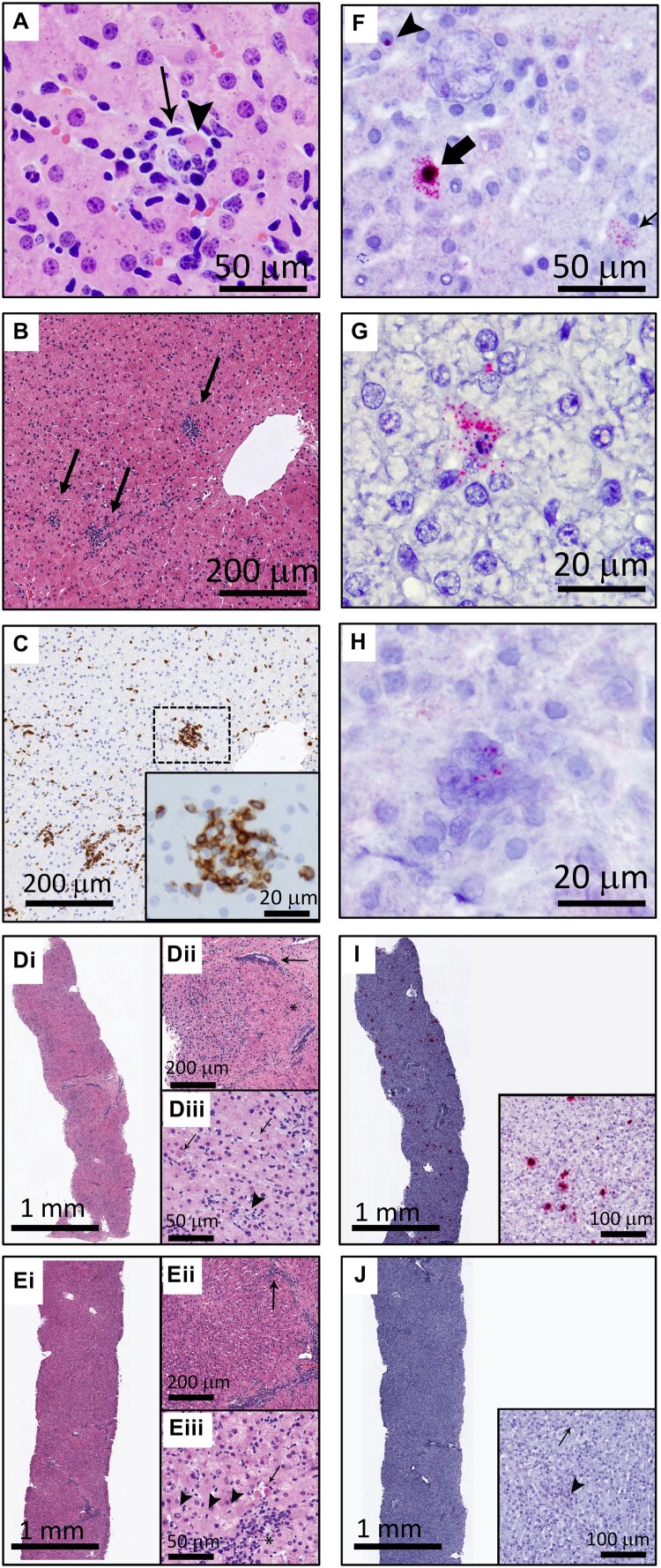
EqPV-H infects hepatocytes and results in hepatocellular necrosis with lymphocytic infiltrates. (A) An individual, necrotic hepatocyte (arrowhead) surrounded by small lymphocytes (arrow). Horse I, HE. (B) Random multifocal clusters of lymphocytes in the hepatic parenchyma (arrows). Horse J, HE. (C) Cells within the clusters of lymphocytes are CD3 positive. Horse J, CD3 IHC. (D) Horse A had the most severe biochemical and clinical hepatitis, which corresponded with the most severe liver pathology. At the beginning of hepatitis (week 7), there was marked increase in cellularity throughout the parenchyma (Di). Pathology was minimal in periportal regions (asterisk, Dii) and portal tracts contained few inflammatory cells (arrow, Dii). Increased numbers of individual necrotic hepatocytes (arrows, Diii), and clusters of lymphocytes, neutrophils, and macrophages surrounding necrotic hepatocytes (arrowhead, Diii), were found throughout zones 2 and 3 of lobules. (E) Seven days later, Horse A’s parenchymal cellularity was already reduced (Ei, Eii). Inflammatory cells and individual necrotic hepatocytes were reduced overall, although portal tracts still contained increased numbers of mixed inflammatory cells (arrow, Eii). Increased numbers of hepatocytes with mitotic figures (arrowheads, Eiii) and fewer individual necrotic cells (arrow, Eiii) were found throughout the parenchyma, compared to the week 7 biopsy (D). Inflammatory cells in portal tracts narrowly breached the limiting plate (asterisk, Eiii). (F) Three hepatocytes are shown demonstrating the range of *in situ* hybridization intensity with punctate nuclear (arrowhead), cytoplasmic (thin arrow), and combined nuclear and cytoplasmic (broad arrow) hybridization. Horse J, EqPV-H ISH, antisense probe. (G) An individual necrotic cell with nuclear karyorrhexis and EqPV-H hybridization within the cytoplasm and extending into the surrounding parenchyma. Horse B, EqPV-H ISH, antisense probe. (H) A focal aggregate of cells in the parenchyma with positive hybridization. Horse I, EqPV-H ISH, sense probe. (I) The biopsy with the most severe pathology had large numbers of hepatocytes with mild to strong positive nuclear and cytoplasmic hybridization (Horse A week 7, same sample as D, EqPV-H ISH, antisense probe). (J) One week later, EqPV-H hybridization was rare (Horse A week 8, same sample as E, EqPV-H ISH, antisense probe) with only mild nuclear (arrowhead) and/or cytoplasmic (arrow) hybridization.

We next wanted to determine which cells in the liver were infected, and to associate infection with pathology. Liver samples from experimentally-inoculated horses during hepatitis (Horses A, B, E, H–J, and L) showed scattered hepatocyte necrosis and mild lymphocytic infiltrate (Figure 4(A), Supplemental Figure 1). Multifocal random clusters of up to 25 lymphocytes were scattered throughout the parenchyma, and portal tracts were infiltrated by small to moderate numbers of lymphocytes that occasionally breached the limiting plate (Figure 4(B)). The majority of these lymphocytes were CD3+ T lymphocytes (Figure 4(C)). The infiltrates were negative for Pax5+ B cells, but were frequently infiltrated by Iba1+ macrophages (Supplemental Figure 2A, B). Horse A, which had clinical signs of liver failure, had the most severe histopathologic changes at the onset of hepatitis (Figure 4(D)). Inflammatory infiltrates and hepatocellular necrosis had a primarily centrilobular distribution, as has been described for Theiler’s disease [2,6]. Evidence of resolution and recovery was already evident 7 days later (Figure 4(E)).

*In situ* hybridization was used to determine whether the pathologic changes were associated with infected cells. EqPV-H NA were detected in small numbers of scattered hepatocytes with signal ranging from individual punctate hybridization in the nucleus to large amounts of hybridization in the nucleus and cytoplasm (Figure 4(F), Supplemental Figure 1). These different viral loads likely reflect the stage of infection in each cell, with large amounts of NA suggestive of active replication within the cell [18]. Necrotic cells and focal cellular aggregates within the parenchyma were positive for EqPV-H NA as well (Figure 4(G,H)). More severe pathology was associated with stronger and more widespread hybridization signal (Horse A, Figure 4(I,J)).

Pre-inoculation liver from three horses (H-J) showed no evidence of hepatitis or EqPV-H-positive cells and post-recovery samples (all liver biomarkers within reference interval) showed markedly reduced number and intensity of EqPV-H positive cells with no clear signs of continuing hepatitis (data not shown). The rare, individual hepatocytes with positive hybridization in the recovered horses most commonly had a single punctate signal in the nucleus with rare cytoplasmic hybridization, which is consistent with the lower viral loads observed in livers >15 weeks after infection (Figure 1(B)). Reticulin and Masson’s trichrome stains were within normal limits in all three horses at all time points tested (Supplemental Figure 2C, D).

Although RNAScope® ISH is designed to target cellular mRNA and does not label cellular DNA due to its secondary chromatin structure, it can label viral DNA in addition to viral RNA. DNase pre-treatment had minimal to no impact on labelling with anti-sense probes, indicating the presence of viral RNA (Supplemental Figure 3). DNase pre-treatment almost completely abolished the labelling with sense probes (which detect anti-sense DNA), confirming the efficacy of DNase treatment (Supplemental Figure 3). Together, these findings indicate that EqPV-H infects and replicates in hepatocytes and viral infection is associated with liver pathology during hepatitis.

### Allogeneic MSC preparations can transmit EqPV-H

Before evaluating routes of natural transmission, we evaluated whether EqPV-H could be transmitted iatrogenically via allogeneic MSC preparations, as was suggested in a recent case series [7]. We tested both IA and intralesional injection into injured SDFT, as these are two common MSC treatment modalities in equine sports medicine [19]. Allogeneic MSC for equine therapeutic use are prepared in various ways that could influence the amount of EqPV-H contamination. Some laboratories culture MSC with FBS as the serum source (MSC-FBS), while others use autologous horse serum to reduce xenogeneic immunogenicity (MSC-AHS) [19].

**Figure 5.**
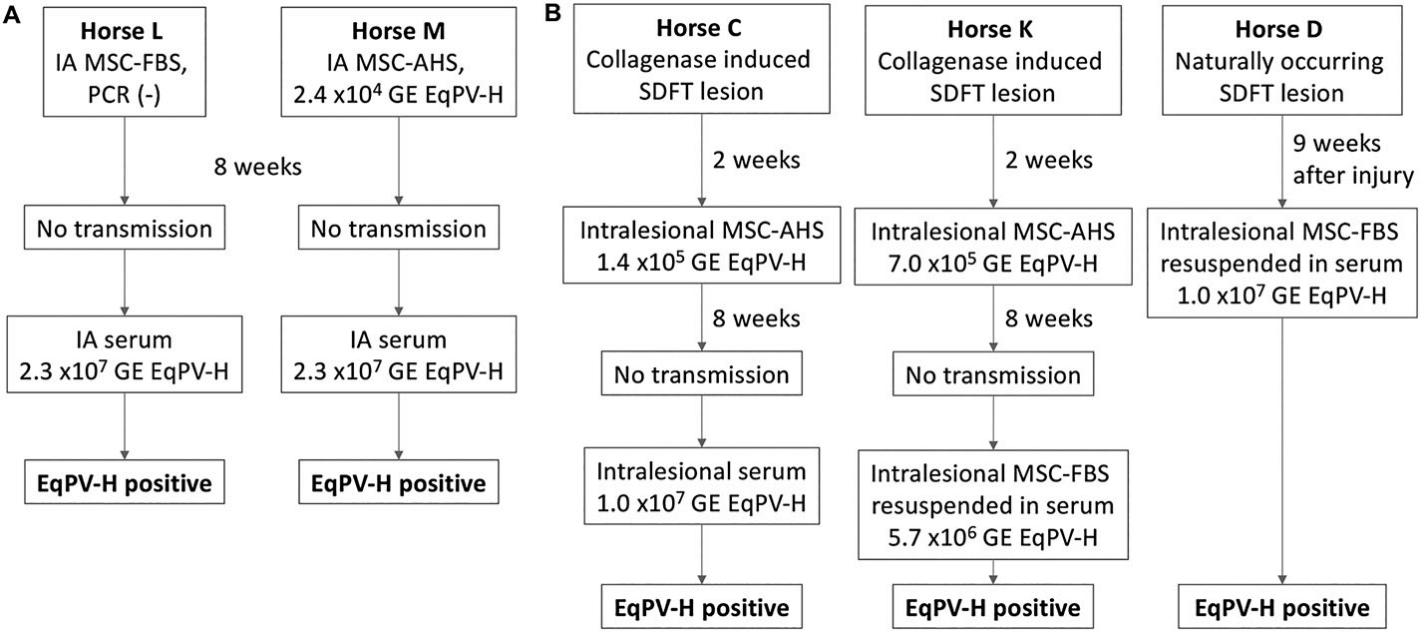
EqPV-H can be transmitted by intra-articular (A) or intra-tendinous (B) injection. Allogeneic bone marrow-derived MSC were collected from a highly EqPV-H viremic horse. Cells were cultured in media containing either foetal bovine serum (MSC-FBS) or EqPV-H^+^ autologous horse serum (MSC-AHS), and washed twice in PBS before re-suspension in 1 ml PBS or EqPV-H^+^ AHS for inoculation. If horses did not become EqPV-H^+^ by 8 weeks, an additional inoculation with a higher viral load was administered, as indicated. SDFT, superficial digital flexor tendon; MSC, mesenchymal stromal cells; FBS, foetal bovine serum; PBS, phosphate-buffered saline; AHS, autologous horse serum; GE, genome equivalents.

No transmission was observed when MSC-FBS or EqPV-H qPCR positive MSC-AHS were administered IA (Figure 5(A)). Subsequent IA inoculation with horse serum to administer a higher EqPV-H dose was successful (Figure 5(A)), indicating that the virus can cross the synovial membrane. Intralesional inoculation of the SDFT with MSC-AHS also failed to transmit EqPV-H (Figure 5(B)), while challenge inoculation with a higher dose in horse serum transmitted the virus (Figure 5(B), Horse C). Based on the observation that horses became viremic after direct injection of EqPV-H-positive horse serum (Horse L, M, C), but not after injection of EqPV-H-positive MSC preparations (Horse M and C), we wanted to exclude the possibility that the presence of MSC could potentially inhibit efficient EqPV-H transmission. Challenge with intralesional MSC-FBS resuspended in high-EqPV-H-titer horse serum successfully transmitted EqPV-H to Horse K, indicating that MSC by themselves do not prevent EqPV-H transmission. This was later confirmed in Horse D which suffered from a naturally occurring SDFT lesion (Figure 5(B)).

Based on these findings, we propose that allogeneic MSC therapy for musculoskeletal injuries has the potential to transmit EqPV-H, especially when the EqPV-H load in these preparations is 5 × 10^6^ GE/ml or higher.

### Vertical EqPV-H transmission was not detected

**Figure 6.**
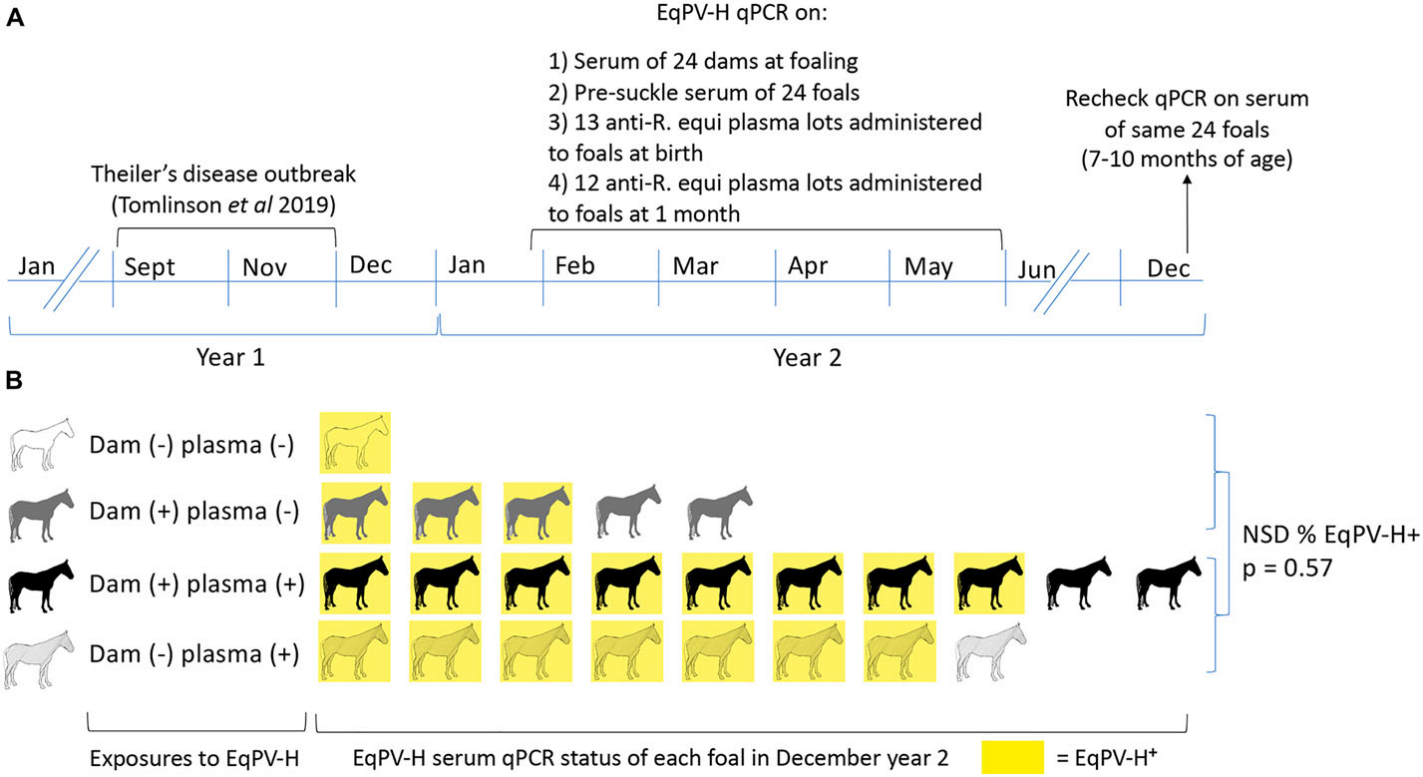
EqPV-H was not transmitted vertically, but was efficiently transmitted to foals by 7–10 months of age. (A) Following an outbreak of Theiler’s disease in September–November of year 1, all foals born on the farm were monitored for EqPV-H infection. EqPV-H qPCR was performed on dam and foal serum collected at birth (ranged from January-May) and foal serum from December. All foals received hyperimmune plasma at birth and at one month of age and these plasma lots were tested by qPCR. (B) Foals were exposed to EqPV-H positive dams and/or plasma and no clear association between these exposures and foal viremia at 7–10 month of age was discernable. Fifteen foals were born to EqPV-H^+^ dams and 9 to EqPV-H^−^ dams, but no *in utero* transmission was observed, as all foals were EqPV-H serum qPCR negative at birth. After birth, foals were exposed to EqPV-H^+^ dams, EqPV-H^+^ hyperimmune plasma, or both (indicated by each row). In December of their birth year, 79% (19/24) of foals were EqPV-H serum qPCR positive (indicated by yellow colour). There was no statistical difference in proportion of infected foals between those that had received EqPV-H^−^ or EqPV-H^+^ plasma (*p* = 0.57). *R. equi*, *Rhodococcus equi*; NSD, no significant difference.

Since many parvoviruses can be transmitted vertically [20,21], we evaluated vertical transmission of EqPV-H on a Standardbred breeding farm which had an outbreak of Theiler’s disease in the fall [2] (Figure 6(A)). The following spring, serum was collected from all 24 mares and foals at birth before they suckled. Aliquots of anti-*R. equi* plasma lots that were administered to the foals at birth and at one month of age were also collected. Foals were again sampled in December of the birth year at 7–10 months of age (Figure 6(A)). Fifteen dams were EqPV-H positive (Ct = 23.9–34.8). All 24 foals were EqPV-H serum qPCR negative before suckling, even when born to an EqPV-H-positive dam (*n* = 15), indicating that vertical transmission *in utero* had not occurred. In December, however, 79% (19/24) of these foals were EqPV-H serum qPCR positive. We hypothesized that likely sources of EqPV-H exposure to foals were (i) contact with EqPV-H-infected dams (horizontal transmission) and/or (ii) hyperimmune anti-*R. equi* plasma administered to foals at 1 d and 1 month of age (equine biologic transmission). Screening of the plasma showed that 11 (44%) of 25 lots used that season were qPCR positive for EqPV-H (Ct = 30.8–36). Using the qPCR of the dams’ serum and *R. equi* plasma lots, we divided the foals into 4 groups based on the exposure types they experienced (Figure 6(B)). EqPV-H prevalence was high in all four groups at 7–10 months of age, indicating efficient transmission, but one particular exposure effect could not be identified. Indeed, Fisher exact test comparison of EqPV-H prevalence in foals that received virus-positive plasma (*n* = 18) versus those that received virus-negative plasma at both times (*n* = 6), irrespective of EqPV-H status of the dam, showed no statistical difference (*p* = 0.57).

### EqPV-H transmission via horse flies could not be demonstrated

Theiler’s disease cases without historical equine biologic product administration typically occur in the late summer to fall, suggesting EqPV-H transmission in the spring and summer [2]. Given this seasonality, and the known possibility of blood transfer [1], we hypothesized mechanical transmission by biting flies was likely. To test this, we used horse flies, which are expected to transmit the largest volume of blood per bite (2 nl) compared to other flies [22].

**Figure 7.**
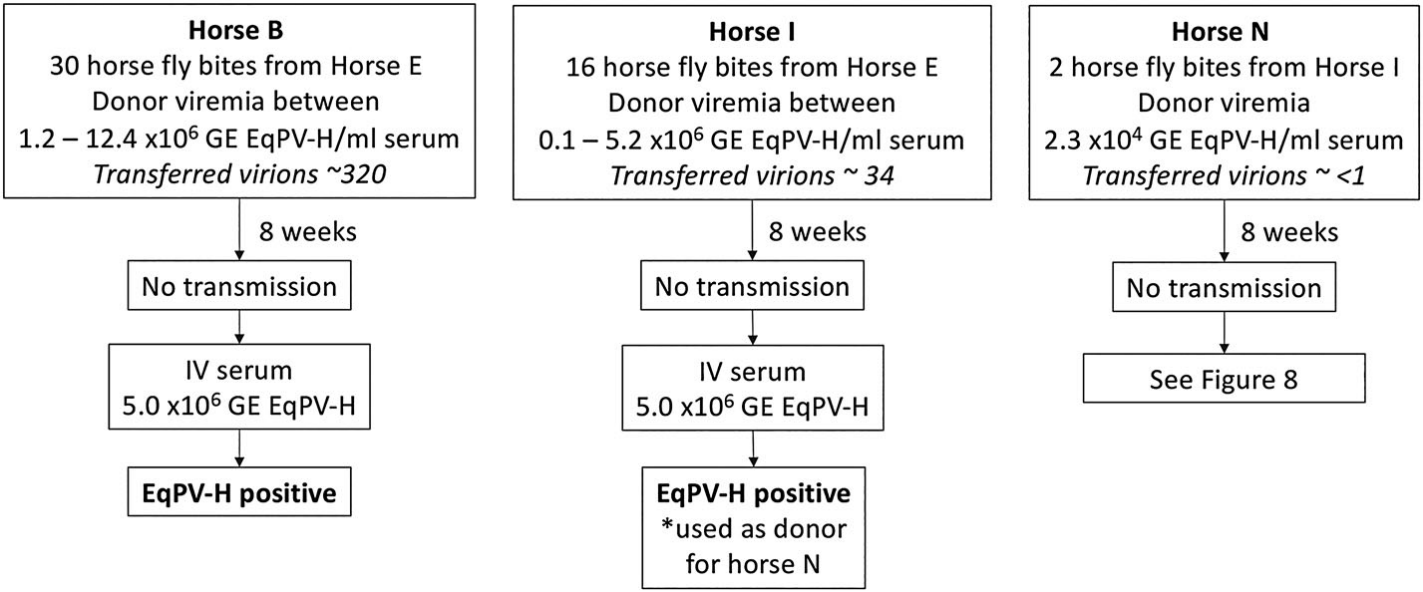
EqPV-H transmission via horse flies could not be demonstrated. Horse flies were fed on EqPV-H viremic horses, dependent on horse fly capture and feeding behaviour. Flies that fed on EqPV-H^+^ donors were immediately transferred to EqPV-H^−^ recipient horses, where they fed to repletion. The estimated number of virions transmitted to each horse was calculated based on donor viremia (GE/ml serum) at the time of each fly bite, 2 nl of blood transfer per bite, and an estimated 40% packed cell volume of the donor (60% serum volume). If horses did not become EqPV-H**^+^** by 8 weeks after fly feeding, an additional inoculation was administered, as indicated, to demonstrate susceptibility to infection. GE, genome equivalents.

We first evaluated the EqPV-H status of flies that had fed on donor horses and found that a single pool of heads from 8 flies and individual abdomens of 4 flies were all EqPV-H qPCR positive. This indicates that horse flies feeding on EqPV-H-positive horses can function as mechanical vectors for this virus and, thus, can potentially transmit EqPV-H to recipient horses. Horse fly transmission attempts were then performed in three donor-recipient pairs, using various numbers of bites. Despite up to 30 transmission bites during high donor viremia, none of the three recipients became infected (Figure 7). All three recipients were subsequently demonstrated susceptible to EqPV-H infection (Figure 7).

### EqPV-H is shed into the environment and oral transmission was demonstrated

**Figure 8.**
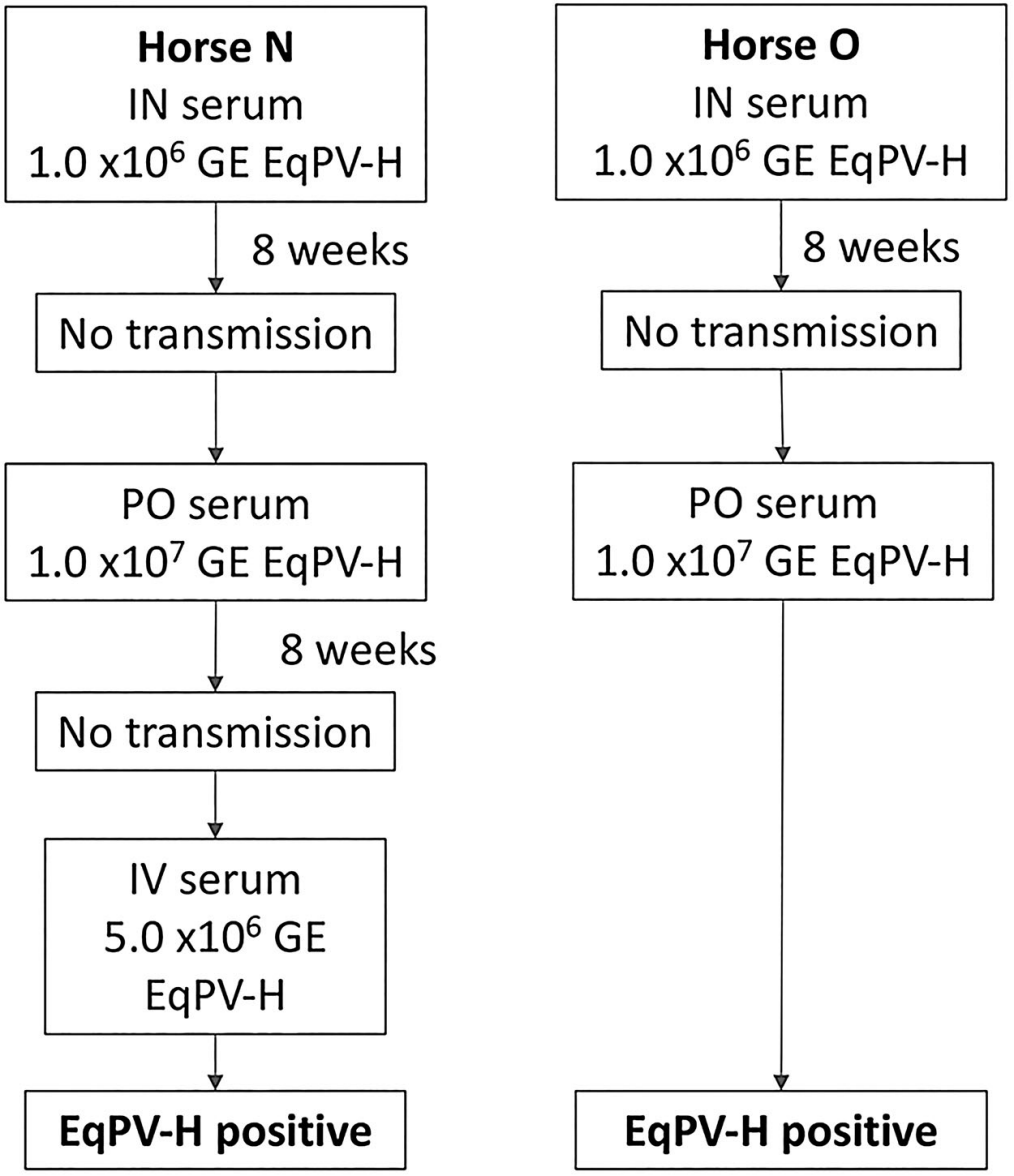
EqPV-H oral transmission was demonstrated. Two horses were first inoculated intranasally (IN) with horse serum containing 1 × 10^6^ GE/ml EqPV-H, but did not become EqPV-H^+^ by 8 weeks. An additional inoculation was performed orally (PO) with horse serum containing 1 × 10^7^ GE/ml EqPV-H. Horse N did not become EqPV-H^+^ by 8 weeks after PO inoculation, therefore an additional inoculation was administered, as indicated. GE, genome equivalents.

We next evaluated natural horizontal transmission modes, such as inhalation or ingestion, which require that virus is shed into the environment. qPCR of nasal, oral, and faecal swabs from 6 EqPV-H experimentally-inoculated horses (Horses A, H-J, L, and M) demonstrated intermittent shedding by each route (Table 1). The shedding period was centred around peak viremia, but shedding could continue at least 10 weeks after inoculation. Based on these findings, we hypothesized that EqPV-H could be transmitted through the upper respiratory tract by nasal contact or inhalation, or through the digestive tract via ingestion. To test this, Horses N and O were inoculated with EqPV-H in serum intranasally, but neither horse became viremic. Subsequently, each horse was inoculated with EqPV-H in serum orally, and Horse O became viremic by week 4 (Figure 8). This finding demonstrates natural transmission of EqPV-H is possible, at least via the oral route, and thus should be considered during the development of control measures for this important equine pathogen.

**Table 1.** EqPV-H is shed in nasal and oral secretions and in faeces. Shedding kinetics (median and range) as determined by qPCR are shown. p.i., post inoculation; GE, genome equivalents; LOQ, limit of quantitation. One horse was still shedding nasally and orally 10 weeks after inoculation at the end of monitoring.

Source	No. shed	Onset (week p.i.)	Duration (weeks)	Peak (GE/swab)	Min serum viremia during shedding (GE/ml)
Nasal	6/6	5.5 (4–6)	3.5 (1– ≥7)	1.59 × 10^4^ (<LOQ–2.87 × 10^6^)	5.59 × 10^4^ (8.94 × 10^2^–4.23 × 10^6^)
Oral	5/6	6 (4–8)	1 (1– ≥ 6)	<LOQ (<LOQ–7.82 × 10^3^)	8.91 × 10^4^ (4.92 × 10^3^–4.23 × 10^6^)
Faecal	6/6	5.5 (4–8)	3 (2–3)	3.13 × 10^3^ (<LOQ–7.09 × 10^3^)	7.67 × 10^4^ (1.89 × 10^4^–2.67 × 10^6^)

## Discussion

In this paper, we provide evidence that EqPV-H, a novel equine parvovirus, is hepatotropic and causes hepatitis. Additionally, we investigated several routes of iatrogenic and natural transmission which could account for the high EqPV-H prevalence observed amongst horses.

Specifically, we demonstrated that EqPV-H infects individual hepatocytes and is associated with hepatocyte necrosis and lymphocytic infiltrates. Although this study is limited by the lack of concurrent mock-inoculated control horses, normal pre-infection serum biochemistry and biopsies served as internal controls. Additionally, the ISH and histopathologic data, combined with kinetic data showing that the onset of hepatitis coincided with seroconversion and a marked reduction in viremia, provide compelling evidence that EqPV-H is a causative agent of hepatitis in horses and is the likely cause of Theiler’s disease. Given the association of EqPV-H with hepatitis and Theiler’s disease demonstrated in previous studies [1,2,7] and further supported by our current work, it is essential to develop infection control strategies. As a first step to control the disease, we must know how it is transmitted beyond iatrogenic blood transmission. Here, we undertook a small survey of 5 routes of transmission, including both iatrogenic and natural modes based on the epidemiology of the disease as reported in recent case series [2,7] as well as our findings of viral shedding into the environment.

We found that EqPV-H can establish systemic infection after IA or intralesional tendon injection with EqPV-H in serum. These sites are frequently treated with allogeneic MSC [19]. However, it took a large viral dose to achieve this, and while equine MSC are sometimes prepared by culture in AHS [19], they are not commonly resuspended in equine serum for injection. Therefore, we suggest the risk of EqPV-H transmission by allogeneic MSC administration is likely low, especially if equine serum is not used in the culture or final preparation of these cells. However, since (i) the minimum infectious dose of EqPV-H is currently unknown, (ii) inter-individual variation in vascularity or inflammation at the injection site could influence distribution and (iii) cases of Theiler’s disease have been reported in horses having received allogeneic MSC treatments 5–8 weeks earlier [7], we suggest that donor horses and/or final allogeneic MSC preparations should be screened for EqPV-H before use.

Based on the findings in our study, vertical transmission does not appear to be a major contributor to the epidemiology of EqPV-H. Specifically, we did not observe vertical transmission among 15 foals born to EqPV-H-positive dams on a farm that had recently suffered an outbreak of Theiler’s disease [2]. In comparison, transmission rates of some vertically transmitted infectious agents in horses include 25% for Equine hepacivirus [23], and 18% for *Neospora hughesii* [24]. Some explanations for the lack of vertical transmission of EqPV-H observed in this study compared to species in which vertical transmission of parvoviruses does occur [20,25], could be the tissue tropism of the specific parvoviruses or the different placentation and antiviral capacities of the host placenta [26,27].

In addition to evaluating vertical transmission, we also observed that routine administration of hyperimmune *R. equi* plasma to foals represents a source of virus introduction to the farm. Additionally, we found that the majority of foals became EqPV-H positive before 1 year of age, despite the fact that Theiler’s disease has never been reported in this age group. This suggests that the lack of susceptibility to severe hepatic necrosis in foals is due to other factors, such as immunologic development, rather than susceptibility to infection. Future studies should, therefore, focus on the immune responses generated upon EqPV-H infection and which immune components are important for controlling disease.

Mechanical transmission of viruses via horse flies has been reported. For equine infectious anaemia and hog cholera, transmission is consistently achieved under 34 bites and often with ≤ 5 bites [28,29]. This is in contrast to bovine leukaemia virus, which can be transmitted by horse flies, but which requires over 50 bites and feeding on a highly viremic donor [30]. Here, we did not observe transmission of EqPV-H with up to 30 horse fly bites. Some explanations for the lack of EqPV-H transmission via horse flies in our study include the (i) low number of bites tested, (ii) ∼15 min delay between feeding on donors and recipients and (iii) low donor viremia resulting in limited number of virions transmitted per bite. Indeed, the number of virions transmitted per bite would have reached ∼60 virions at best. Additionally, we did not have a concurrent positive control of transmission of another virus, such as equine infectious anaemia, to demonstrate the adequacy of our technique. We, therefore, conclude that mechanical transmission of EqPV-H via biting flies cannot be ruled out based on this study, but suggest this method of spread is likely to be inefficient and require a large number of bites. Regardless, vector-borne transmission of EqPV-H must still be considered likely since non-biologic-associated cases of Theiler’s disease typically occur in late summer and fall [2].

We found that horses shed EqPV-H into the environment via nasal and oral secretions and in faeces, as has been described for other parvoviruses such as canine parvovirus-2 and porcine parvovirus [31,32]. While the amount of EqPV-H shed was typically low, large amounts of virus, e.g. 2.9 × 10^6^ GE in a single nasal swab, were occasionally detected. Parvoviruses are notoriously hardy viruses [33,34] and, thus, even low amounts of shedding could accumulate in the environment to create substantial contamination. Importantly, we could demonstrate that oral administration of EqPV-H resulted in a successful infection in one horse. Additional studies are needed to determine the relevance of this finding in the epidemiology of the virus, including (i) more horses to better evaluate the efficiency of transmission via this route and (ii) different viral loads for inoculation to assess the minimal infectious dose.

In summary, this study demonstrates that EqPV-H is hepatotropic and causes hepatitis. Allogeneic MSC preparations with high viral load can transmit EqPV-H, and thus, donor horses and/or final products should be confirmed EqPV-H-free before use. Moreover, horizontal transmission of EqPV-H is common among horses, horses shed EqPV-H via multiple routes, and EqPV-H can be transmitted orally. While vertical transmission, horse fly transmission, and nasal transmission were not demonstrated in the current study, additional experimental infection studies are needed to determine whether this reflects inefficient or complete lack of transmission. Additional studies on farm transmission of EqPV-H are needed to strengthen farm biosecurity recommendations in the face of Theiler’s disease cases.

## Supplementary Material

Supplemental Material
